# Supplementary material for: The Role of Dishware Size in the Perception of Portion Size in Children and Adolescents with Obesity
Source: Nutrients. 2021 Jun 16;13(6):2062. doi: 10.3390/nu13062062 (PMC8235649; doi:10.3390/nu13062062)
Supplement: Supplementary file 1 [file nutrients-13-02062-s001.zip › nutrients-1238142-supplementary.pdf]

Supplementary Material

Supplementary Table S1: Reasons for the intuitive evaluation for drinking glasses and dishes

Dishware item	Reasons	Big Thirst NW n (%)	Big Thirst OBE n (%)	Big Thirst p	Small Thirst NW n (%)	Small Thirst OBE n (%)	Small Thirst p
**Glass Pair 1**	*No specific reason*	1 (3.7)	12 (20.0)	.13	10 (37.0)	16 (27.1)	.76
	Easy handling, habit	3 (11.1)	8 (13.3)		5 (18.5)	12 (20.3)	
	Volume/amount smaller	0 (0.0)	2 (3.3)		12 (44.4)	29 (49.2)	
	Volume/amount larger	23 (85.2)	38 (63.3)		0 (0.0)	2 (3.4)	
**Glass pair 2**	*No specific reason*	5 (18.5)	10 (17.2)	.84	6 (23.1)	18 (31.0)	.40
	Easy handling, habit	5 (18.5)	7 (12.1)		6 (23.1)	7 (12.1)	
	Volume/amount smaller	2 (7.4)	5 (8.6)		13 (50.0)	26 (44.8)	
	Volume/amount larger	15 (55.6)	36 (62.1)		1 (3.8)	7 (12.1)	
**Glass pair 3**	*No specific reason*	6 (23.1)	14 (24.6)	.63	9 (34.6	17 (29.3)	.85
	Handiness, habit	1 (3.8)	4 (7.0)		1 (3.8	5 (8.6)	
	Volume/amount smaller	2 (7.7)	1 (1.8)		16 (61.5	34 (58.6)	
	Volume/amount larger	17 (65.4)	38 (66.7)		0 (0.0	2 (3.4)	
**Dish Pair 1**	*No specific reason*	4 (14.8)	14 (26.4)	.14	9 (33.3)	16 (30.2)	.66
	Easy handling, habit	2 (7.4)	4 (7.5)		3 (11.1)	3 (5.7)	
	Volume/amount smaller	6 (22.2)	3 (5.7)		15 (55.6)	34 (64.2)	
	Volume/amount larger	15 (55.6)	32 (60.4)		0 (0.0)	0 (0.0)	
**Dish pair 2**	*No specific reason*	7 (25.9)	9 (17.0)	.60	9 (34.6)	25 (47.2)	.32
	Easy handling, habit	2 (7.4)	3 (5.7)		1 (3.8)	0 (0.0)	
	Volume/amount smaller	0 (0.0)	3 (5.7)		16 (61.5)	27 (50.9)	
	Volume/amount larger	18 (66.7)	38 (71.4)		0 (0.0)	1 (1.9)	
**Dish pair 3**	*No specific reason*	8 (29.6)	13 (24.5)	.97	13 (48.1)	18 (34.0)	.16
	Easy handling, habit	1 (3.7)	3 (5.7)		0 (0.0)	3 (5.7)	
	Volume/amount smaller	1 (3.7)	3 (5.7)		13 (48.1)	32 (60.4)	
	Volume/amount larger	17 (63.0)	34 (64.2)		1 (3.7)	0 (0.0)	

All data presented as absolute incidence (percent); Statistics: Chi-Square Tests; Abbreviations: n = absolute incidence; NW = children and adolescents with normal weight; OBE = children and adolescents with overweight and obesity; NW and OBE aged 9 – 17; p = p-value.

Supplementary Table S2: Quantitative cognitive evaluation (qCE) for drinking glasses and dishes

Dishware item	NW/OBE	Med (%)	IQR (%)	p
Glass Pair 1	NW	20.0 (16.7)	19.8 – 20.0 (16.5 – 16.7)	.57
	OBE	20.0 (16.7)	16.3 – 20.0 (13.6 – 16.7)	
Glass pair 2	NW	0.0 (0.0)	-58.2 – 0.0 (-23.28 – 20.91)	.80
	OBE	0.0 (0.0)	-25.1 – 0.0 (-2.48 – 0.0)	
Glass pair 3	NW	39.4 (13.1)	-20.0 – 50.0 (-6.7 – 16.7)	.11
	OBE	0.2 (0.1)	-40.4 – 34.6 (-13.4 – 11.5)	
Dish Pair 1	NW	22.5 (12.5)	-30.0 – 30.0 (-16.7 – 16.7)	.28
	OBE	30.0 (16.7)	-10.5 – 30.0 (-5.8 – 16.7)	
Dish pair 2	NW	0.0 (0.0)	0.0 – 0.0 (0.0 – 0.0)	1.00
	OBE	0.0 (0.0)	-5.6 – 8.4 (-5.6 – 8.4)	
Dish pair 3	NW	14.8 (11.4)	-22.5 – 30.0 (-17.3 – 23.1)	**.031**
	OBE	30.0 (23.1)	15.7 – 30.0 (12.1 – 23.1)	

All data presented as absolute Median (percent); Mann-Whitney-U test; Abbreviations: med = Median; IQR = Interquartile range; NW = children and adolescents with normal weight (Glasses: n = 27; Plates: n = 26); OBE = children and adolescents with overweight and obesity (Glasses: n = 54; Plates: 51); NW and OBE aged 9 – 17; p = p-value.

Supplementary resources 3 – 6 represent the post-hoc analyses of different age groups.

Supplementary Table S3: Results of the intuitive and cognitive evaluation for drinking glasses with participants aged 11 – 17

		**Intuitive evaluation (IE)**						**Cognitive evaluation (CE)**				
**Pair#**	**Glass (content)**	Big thirst n (%)		p	Small thirst n (%)		p	Correct n (%)		False n (%)		p
		NW	OBE		NW	OBE		NW	OBE	NW	OBE	
1	Wide parabolic (120ml)	25 (92.6)	39 (69.6)	**.020**	8 (29.6)	12 (21.8)	.439	7 (25.9)	13 (23.6)	20 (74.1)	42 (76.4)	.821
	High parabolic (100ml)	2 (7.4)	17 (30.4)		19 (70.4)	43 (78.2)						
2	Small wide (250ml)	16 (59.3)	35 (64.8)	.625	6 (23.1)	18 (33.3)	.348	12 (44.4)	37 (69.8)	15 (55.6)	16 (20.2)	**.028**
	Wide (250ml)	11 (40.7)	19 (35.2)		20 (76.9)	36 (66.7)						
3	High narrow (300ml)	23 (88.5)	46 (86.8)	.834	10 (38.5)	10 (18.9)	.060	16 (59.3)	43 (81.1)	11 (40.7)	10 (18.9)	**.036**
	Narrow (250ml)	3 (11.5)	7 (13.2)		16 (61.5)	43 (81.1)						

Data presented as absolute incidence (percent); Statistics: Chi-Square Tests; Abbreviations: n = absolute incidence; NW = children and adolescents with normal weight; OBE = children and adolescents with overweight and obesity; p = p-value.

Supplementary Table S4: Results of the intuitive and cognitive evaluation for drinking glasses with participants aged 10 – 14

		**Intuitive evaluation (IE)**						**Cognitive evaluation (CE)**				
**Pair#**	**Glass (content)**	Big thirst n (%)		p	Small thirst n (%)		p	Correct n (%)		False n (%)		p
		NW	OBE		NW	OBE		NW	OBE	NW	OBE	
1	Wide parabolic (120ml)	25 (92.6)	32 (68.1)	**.016**	8 (29.6)	10 (21.7)	.450	7 (25.9)	10 (21.3)	20 (74.1)	37 (78.7)	.647
	High parabolic (100ml)	2 (7.4)	15 (31.9)		19 (70.4)	36 (78.3)						
2	Small wide (250ml)	16 (59.3)	27 (58.7)	.962	6 (23.1)	16 (34.8)	.300	12 (44.4)	30 (66.7)	15 (55.6)	15 (33.3)	.064
	Wide (250ml)	11 (40.7)	19 (41.3)		20 (76.9)	30 (65.2)						
3	High narrow (300ml)	23 (88.5)	38 (84.4)	.639	10 (38.5)	8 (17.8)	.054	16 (59.3)	37 (82.2)	11 (40.7)	8 (17.8)	**.032**
	Narrow (250ml)	3 (11.5)	7 (15.6)		16 (61.5)	37 (82.2)						

Data presented as absolute incidence (percent); Statistics: Chi-Square Tests; Abbreviations: n = absolute incidence; NW = children and adolescents with normal weight; OBE = children and adolescents with overweight and obesity; p = p-value.

Supplementary Table S5: Results of the intuitive and cognitive evaluation for dishes with participants aged 11 – 17

**Pair#**	**Dishes (content)**	**Intuitive evaluation (IE)** Big hunger n (%)		p	Small hunger n (%)		p	**Cognitive evaluation (CE)** Correct n (%)		False n (%)		p
		NW	OBE		NW	OBE		NW	OBE	NW	OBE	
1	Big bowl (180g)	13 (51.9)	29 (58)	.407	2 (7.4)	12 (24)	.072	14 (51.9)	24 (48)	13 (48.1)	26 (52)	.747
	Small bowl (150g)	14 (48.1)	21 (42)		25 (92.6)	38 (76)						
2	Big plate (100g)	23 (85.2)	37 (74)	.259	8 (29.6)	10 (20)	.345	17 (63.0)	24 (48)	10 (37.0)	26 (52)	.209
	Small plate (100g)	4 (14.8)	13 (26)		19 (70.4)	40 (80)						
3	Big plate (130g)	23 (85.2)	35 (70)	.140	6 (22.2)	10 (20.8)	.888	14 (53.8)	13 (26)	12 (46.2)	37 (74)	**.016**
	Small plate (100g)	4 (14.8)	15 (30)		21 (77.8)	38 (79.2)						

All data presented as absolute incidence (percent); Statistics: Chi-Square Tests; Abbreviations: n = absolute incidence; NW = children and adolescents with normal weight; OBE = children and adolescents with overweight and obesity; p = p-value.

Supplementary Table S6: Results of the intuitive and cognitive evaluation for dishes with participants aged 10 – 14

**Pair#**	**Dishes (content)**	**Intuitive evaluation (IE)** Big hunger n (%)		p	Small hunger n (%)		p	**Cognitive evaluation (CE)** Correct n (%)		False n (%)		p
		NW	OBE		NW	OBE		NW	OBE	NW	OBE	
1	Big bowl (180g)	13 (51.9)	24 (58.5)	.400	2 (7.4)	10 (24.4)	.072	14 (51.9)	21 (51.2)	13 (48.1)	20 (48.8)	.959
	Small bowl (150g)	14 (48.1)	17 (41.5)		25 (92.6)	31 (75.6)						
2	Big plate (100g)	23 (85.2)	30 (73.2)	.242	8 (29.6)	8 (19.5)	.336	17 (63.0)	19 (46.3)	10 (37.0)	22 (53.7)	.179
	Small plate (100g)	4 (14.8)	11 (26.8)		19 (70.4)	33 (80.5)						
3	Big plate (130g)	23 (85.2)	27 (65.9)	.077	6 (22.2)	10 (25)	.794	14 (53.8)	10 (24.4)	12 (46.2)	31 (75.6)	**.014**
	Small plate (100g)	4 (14.8)	14 (34.1)		21 (77.8)	30 (75)						

All data presented as absolute incidence (percent); Statistics: Chi-Square Tests; Abbreviations: n = absolute incidence; NW = children and adolescents with normal weight; OBE = children and adolescents with overweight and obesity; p = p-value.

Supplementary Text S7: ***Post hoc analyses of the CE score across dishware pairs***

Summary of drinking glasses. Results remained when excluding the four youngest children aged between 9 and 10 years (age range 11 – 17 years; OBE: 57.8% correct; NW: 43.2% correct; X^2 (1, N=242)=4.58; p=.032). When analyzing 10- to 14-year-olds, there were no significant differences between NW and OBE in the CE score across the three pairs of drinking glasses (OBE: 56.2% correct; NW: 43.2% correct; X^2 (1, N=218)=3.44; p=.064).

Summary of dishes. The results remained the same after excluding the four youngest children aged between 9 and 10 (age range 11 – 17 years; OBE: 40.1% correct; NW: 56.0% correct; X^2 (1, N=230)=5.01; p=.024) and when separately analyzing the group of 10- to 14-year-olds (OBE: 40.7% correct; NW: 56.3% correct; X^2 (1, N=203)=4.74; p=.030).
